# Effect of Vision and Surface Slope on Postural Sway in Healthy Adults: A Prospective Cohort Study

**DOI:** 10.3390/life14020227

**Published:** 2024-02-05

**Authors:** Masoud Aghapour, Nadja Affenzeller, Christian Peham, Christiane Lutonsky, Alexander Tichy, Barbara Bockstahler

**Affiliations:** 1 Section of Physical Therapy, Small Animal Surgery, Department for Companion Animals and Horses, University of Veterinary Medicine, 1210 Vienna, Austria; nadja.affenzeller@vetmeduni.ac.at (N.A.); christiane.lutonsky@vetmeduni.ac.at (C.L.); barbara.bockstahler@vetmeduni.ac.at (B.B.); 2 Clinical Unit of Internal Medicine Small Animals, Department for Companion Animals and Horses, University of Veterinary Medicine, 1210 Vienna, Austria; 3 Movement Science Group, Equine Surgery, Department for Companion Animals and Horses, University of Veterinary Medicine, 1210 Vienna, Austria; christian.peham@vetmeduni.ac.at; 4 Platform Bioinformatics and Biostatistics, Department for Biomedical Services, University of Veterinary Medicine, 1210 Vienna, Austria; alexander.tichy@vetmeduni.ac.at

**Keywords:** postural stability, balance control, center of pressure, inclined surface, posturography, body oscillation

## Abstract

Postural stability requires an interaction between cognitive, perceptual, sensory, and motor functions. Thus, impairment in any of these systems may affect postural balance. This study assessed the effect of visual input and surface slope on postural stability. The study was conducted on healthy participants, 11 females and 11 males who were 24–34 years of age. They were asked to perform still upright bipedal standing on flat and +/−20° sloped surfaces with eyes open (EO) and closed (EC). Six center of pressure (COP) parameters were measured by posturography. A significant relationship was observed between COP parameters, standing conditions, and body mass index. Gender had no significant effect on the COP. The loss of visual input within each standing condition did not affect the COP parameters. In contrast, differences were observed between standing on a flat surface and uphill with EC and between standing on a flat surface and downhill with EC and EO. When the participants were standing on inclined surfaces, the loss of vision significantly increased the postural instability. Young healthy adults demonstrated the greatest difficulty in standing uphill with EC. This was followed by standing downhill with EC and standing downhill with EO.

## 1. Introduction

Motor activities and postural control require a complex interaction between cognitive, perceptual, sensory, and motor functions [1,2,3,4]. The generic definition of balance refers to the ability of the body to prevent falling by the tonic activation of muscles, considering both antigravity support and maintaining the center of gravity (COG) of the body or center of mass (COM) within base of support (BOS) of the body with the least possible sway [1,4,5]. Postural balance is achieved through dynamic and static balance, requiring coordination among the central nervous system and the visual, somatosensory, and vestibular systems [1,3,4,5,6]. This coordination results in antigravity support from posture-stabilizing muscles in the legs, trunk, and neck [3,4].

The gold standard for the laboratory measurement of postural stability is called posturography, which includes locating the center of pressure (COP) within the BOS of a subject while standing still [1,7]. Posturography can be performed under various aggravating test conditions to investigate the effects on postural control [7,8,9,10]. Posturographic measurements are based on the measurement of vertical ground reaction forces [1]. The vertical ground reaction forces represent the sum of all vertical forces between a physical object and its contact surface. The COP is the point at which the instantaneous vector of the vertical ground reaction forces is applied [1,11]. The displacement of the COP is an indirect measure of the functionality of postural control and thus a measure of the ability to maintain balance. Various COP parameters can be measured using posturography, such as the displacement of the COP in the anterior–posterior and mediolateral directions, total length of the excursion of the COP, average speed (AS) of the COP, support surface (SS) of the COP, and SS per unit length of the COP [1,11,12]. These conventional (linear) COP parameters are indicators of postural stability [13,14]. In addition to these traditional measures, in recent years, non-linear methods such as sample entropy and approximate entropy have been investigated [15]. These non-linear algorithms estimate the randomness of data series and assess the non-linearity within postural sway dynamics by evaluating irregularities present in the COP’s time series data [15,16].

Postural stability is affected by the size and shape of the BOS; height of the COM; relationship between the line of gravity and BOS; friction on the support; segmental alignment of the body (mass distribution); and visual, psychological, and physiological factors [17]. Hence, various cognitive, sensory, or motor impairments can cause a patient to experience difficulties in maintaining, achieving, or restoring equilibrium. Increased postural instability may be an indicator of decreased neuromuscular control during aging [2,3,18,19,20,21]. Postural instability may also be due to neurological diseases, such as Alzheimer’s disease [10,22,23,24] or Parkinson’s disease [25,26]. Furthermore, musculoskeletal disorders, such as injury to the anterior cruciate ligament [27], injuries to the ankle [28,29,30], hip fractures [31], or even head trauma [32,33], can affect postural stability.

Previous studies emphasized the relationship between postural stability and anthropometric parameters, such as weight and height [34,35,36,37,38,39]. For example, body mass index (BMI) is reported to be an important anthropometric parameter influencing postural balance [34,35,36,39] and is classed as a risk factor for falling among seniors [38,40]. In general, weight is considered to be a more significant anthropometric factor than height, although the impact of increased body height on postural balance should not be overlooked [34,36,37,39]. In addition to anthropometric parameters, age [34,41]; gender [36,37]; and visual, vestibular, or proprioceptive impairments [42,43,44,45,46,47,48] have been reported to affect postural balance.

The ability to maintain balance is dependent on the input information from visual, vestibular, and proprioceptive systems; impairments (such as the absence of visual input) in any of these systems can negatively affect postural stability [34,49]. For example, with EC, more considerable body sway was observed among subjects standing upright on a flat surface than among those standing with eyes open (EO) [45]. Furthermore, several studies confirmed the effect of vision on postural stability during an upright stance on a sloped (inclined and declined) surface [50,51,52]. However, contradicting results were reported among young athletes; the degree of visual impairment was not related to postural stability [39], and conventional COP parameters demonstrated a similar amount of balance control [53]. In addition, the EC condition did not affect young football players’ balance when standing on different surfaces (solid vs. foam), while the more demanding single-leg stance condition did [54].

Hence, despite the demonstrated effect of vision and inclined surfaces on postural balance [50,51,52], this influence could not be consistently confirmed in other studies. It appears that this is particularly true for healthy young adults and athletes [35,36,39]. These conflicting results raise the question of whether human postural control mechanisms are challenged differentially by certain environmental conditions, as well as whether an increase in the complexity of test conditions leads to different compensation mechanisms in the stabilization of the body. Changes in feedback mechanisms such as the preference for somatosensory and vestibular sensory information when, e.g., visual feedback is lost, may explain the different outcomes when analyzing postural stability under differing circumstances in the above literature. Indeed, a recent study found that horseback riders seem to rely more on vision when their somatosensory system was challenged than judokas when tested under identical conditions [16]. Based on this literature, postural control is affected differently by various aggravating test conditions. To date, no study has investigated the effect of both vision (EO/EC) and slope (inclined/declined) through an extensive panel of conventional COP parameters.

Hence, this study aimed to investigate linear COP parameters in young healthy adults under different test conditions, when standing on steeply inclined and declined surface slopes under both EO and EC conditions. The following were hypothesized: (1) The COP values for inclined and declined bipedal upright postures are higher compared with those for flat ground; the increase in COP values implies greater changes in the location of the COP [9,52]. (2) Postural balance decreases with EC, most notably when the subject is standing on a sloped surface.

## 2. Materials and Methods

### 2.1. Sample and Procedure

In this prospective cohort study, 22 healthy young adults (11 women and 11 men) were recruited. The sample size was determined on the basis of comparable studies [52,55,56,57]. All participants were free of any musculoskeletal, neurological, and visual disorders. The participants were 24–34 years old (30.3 ± 2.9 years old); their height and body weight ranges were 152–193 (175.1 ± 11) cm and 50–105 (71.8 ± 17.7) kg, respectively. Furthermore, BMI (participant weight in kilograms divided by the square of height in meters) was calculated. The BMI ranges of the female and male groups were 18.4–24.2 (20.8 ± 1.9) and 20.7–28.7 (25.4 ± 2.8) kg/m^2^, respectively. The overall BMI range of participants was 18.4–28.7 (23.1 ± 3.3) kg/m^2^.

The measurements were performed in a standing position (i.e., upright bipedal standing) on flat ground, in an uphill condition with a +20° slope, and in a downhill condition with a −20° slope. The determination of this angle was based on previous investigations [9,58]. All measurements were performed with EO and EC. Three consecutive measurements, each lasting 30 s, were performed [59]. The standing conditions were as follows:

- C1: standing on flat ground with EO (standard condition);
- C2: standing on flat ground with EC;
- C3: standing uphill with EO;
- C4: standing uphill with EC;
- C5: standing downhill with EO;
- C6: standing downhill with EC.

The order of conditions was randomized prior to the study; however, the EO condition was always followed by the EC condition. The participants were requested to stand still on a pressure measurement platform under different conditions with both arms at their sides. For normal standing, the feet must align with the shoulders while focusing (EO) or pretending to focus (EC) on a spot on a wall. All participants were barefoot during the measurements.

Measured data were analyzed with the custom-made software named Pressure Ana-lyzer (Michael Schwanda, version 4.8.5.0). Each trial was immediately evaluated. A valid trial was confirmed if the participant maintained the correct position as instructed. Trials in which the participant had the least body oscillation for 20 s were selected for further analysis. For a trial to be valid, a participant should silently stand still with the feet aligned with the shoulders and arms at the sides without head and hand movements while focused on a straight direction. The result of each trial was exported as Excel data (Microsoft Excel 2016). Then, the average of three measurements under each condition was calculated.

### 2.2. Approval and Consent

This study was performed in accordance with the Declaration of Helsinki and discussed and approved by the institutional ethics committee of the Medical University of Vienna in accordance with good scientific practice guidelines and national legislation (EK No. 2261/2021). All participants provided written consent prior to the study.

### 2.3. Equipment

**Figure 1:**
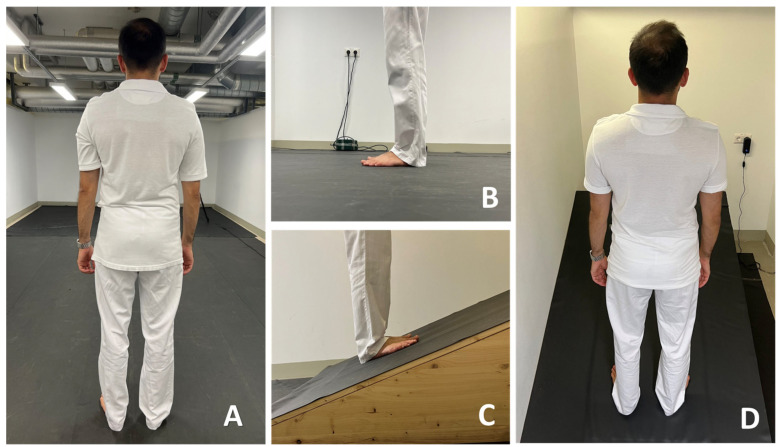
Measurement of ground reaction forces on flat and sloped surfaces: (**A**,**B**) measurement platform in normal standing position and (**C**,**D**) measurement platform embedded in 20° slope.

Posturography was performed using two measurement platforms. Measurements in a standing position on flat ground were performed using a Zebris platform (FDM Type 2, Zebris Medical GmbH, Allgäu, Germany) equipped with 15,360 sensors covering an area of 203 × 54.2 cm; the measuring frequency was 100 Hz (Figure 1A). Uphill/downhill measurements were performed using another Zebris platform (FDM Type 1.5, Zebris Medical GmbH, Allgäu, Germany) equipped with 11,264 sensors covering an area of 149 × 54.2 cm; the measuring frequency was also 100 Hz. The shorter measurement platform was placed on a 20° slope to simulate uphill/downhill conditions (Figure 1B). The measurement range (1–120 N/cm^2^), accuracy (±5% (FS (full scale))), and hysteresis (<3% (FS)) between the two platforms were reported to be the same. The sensor size of both platforms was 0.72 × 0.72 cm. To standardize the coefficient of friction, the pressure plate was covered with a black 1 mm thick non-slip rubber mat made of polyvinyl chloride. All measurement procedures were filmed using a Panasonic NV-MX500 camera (Panasonic, Kadoma, Osaka, Japan), and data were gathered using the custom-made software “Pressure Analyzer” (Michael Schwanda, version 4.8.5.0).

### 2.4. COP Parameters and Romberg Index

In this study, the following body COP parameters were measured:

- Mediolateral displacement (MLD) in millimeters.
- Anterior–posterior displacement (APD) in millimeters.
- Total length (L) in meters; defined as the total excursion of the COP during the measurement time calculated by summing the distance among the successive locations of the COP.
- AS in millimeters per second; defined as the mean speed of the COP movement.
- SS in square millimeters; area of the ellipse containing 90% of all COP points.
- Quotient of SS in square millimeters per unit length in millimeters (LFS).

The Romberg index (RI) is the ratio of EC score to EO score multiplied by 100 (EC/EO × 100) [60]. This index reflects the contribution of vision to maintain posture [57,60,61]. A high RI indicates a high visual information contribution to the variable; thus, the instability is considerable. In this study, the RI was calculated for each standing condition, as follows:

- $RI\;1=\frac{standing\;on\;flat\;ground\;with\;EC}{standing\;on\;flat\;ground\;with\;EO}\times 100$
- $RI\;2=\frac{standing\;uphill\;with\;EC}{standing\;uphill\;with\;EO}\times 100$
- $RI\;3=\frac{standing\;downhill\;with\;EC}{standing\;downhill\;with\;EO}\times 100$

### 2.5. Statistical Analysis

The statistical analysis of the measured data was performed using IBM SPSS version 27 (IBM, Chicago, IL, USA). Statistical significance was assumed with an alpha level of *p* < 0.05. The normal distribution of parameters was evaluated by the Shapiro–Wilk test, and log10 transformation was performed for variables without normal distribution. Descriptive statistics were calculated for each COP parameter under each condition and RI. Linear mixed model analysis was implemented to assess any relationship between COP parameters and standing condition, gender, and BMI. Furthermore, pairwise comparisons with Bonferroni’s alpha corrections were performed to evaluate the relationship between each COP parameter, measurement condition, and RI.

## 3. Results

### 3.1. Descriptive Statistics

In general, when comparing our standard condition (C1) with other conditions, all COP parameters of C4 increased and differed significantly from C1. Furthermore, APD, L, and AS of C6 increased and were significantly different than C1 (Table 1). Detailed results of the pairwise comparisons between the standing condition and COP parameters with Bonferroni’s alpha corrections are presented in the Supplementary Materials (Table S1).

### 3.2. Relationship between COP Parameters and Standing Conditions, Gender, and BMI

A significant relationship between the standing conditions and all COP parameters was observed in this study (*p* ≤ 0.009). Gender had no significant effect; however, the BMI had a considerable influence on all COP parameters (*p* ≤ 0.001), except on MLD and SS.

### 3.3. Pairwise Comparison of Standing Conditions

The loss of visual input did not significantly affect the COP parameters under each standing condition. In comparison with C1, standing on an inclined surface (uphill) with EO did not affect stability. In contrast, the absence of visual input (i.e., EC) while a subject stood uphill increased body sway, and all COP values increased (*p* ≤ 0.048). While standing downhill with EO, the amplitude of the mediolateral and anterior–posterior fluctuations did not change significantly compared with C1; however, L and AS increased (*p* ≤ 0.017). The absence of visual input (EC) while standing downhill led to an additional increase in displacement in the anterior–posterior direction (*p* ≤ 0.001).

#### 3.3.1. C1 vs. Other Standing Conditions

The pairwise comparisons of the COP parameters between C1 and C2 and between C1 and C3 showed no significant variations. All the COP parameters between C1 and C4 differed in this study (*p* ≤ 0.048). The difference in COP parameters between C1 and C5 was limited to L and AS (*p* ≤ 0.017). However, APD, L, and AS differed between C1 and C6 (*p* ≤ 0.001).

#### 3.3.2. C2 vs. Other Standing Conditions

In terms of MLD, APD, SS, and LFS, C2 differed from C4 (*p* ≤ 0.013). No significant differences were observed between C2 and the other remaining conditions.

#### 3.3.3. C3 vs. Other Standing Conditions

In terms of L and AS, C3 differed with C6 (*p* ≤ 0.032). No other differences were observed between C3 and other standing conditions.

#### 3.3.4. C4 vs. Other Standing Conditions

As reported, C4 differed with C1 in terms of all COP parameters and with C2 in terms of MLD, APD, SS, and LFS. No significant differences were observed between C4 and C3 and between C4 and C6. The difference between C4 and C5 was limited to APD (*p* = 0.046).

#### 3.3.5. C5 vs. Other Standing Conditions

In addition to the difference between C5 and C1 and between C5 and C4, as previously reported, no other differences were observed between C5 and the other remaining conditions.

#### 3.3.6. C6 vs. Other Standing Conditions

**Figure 2:**
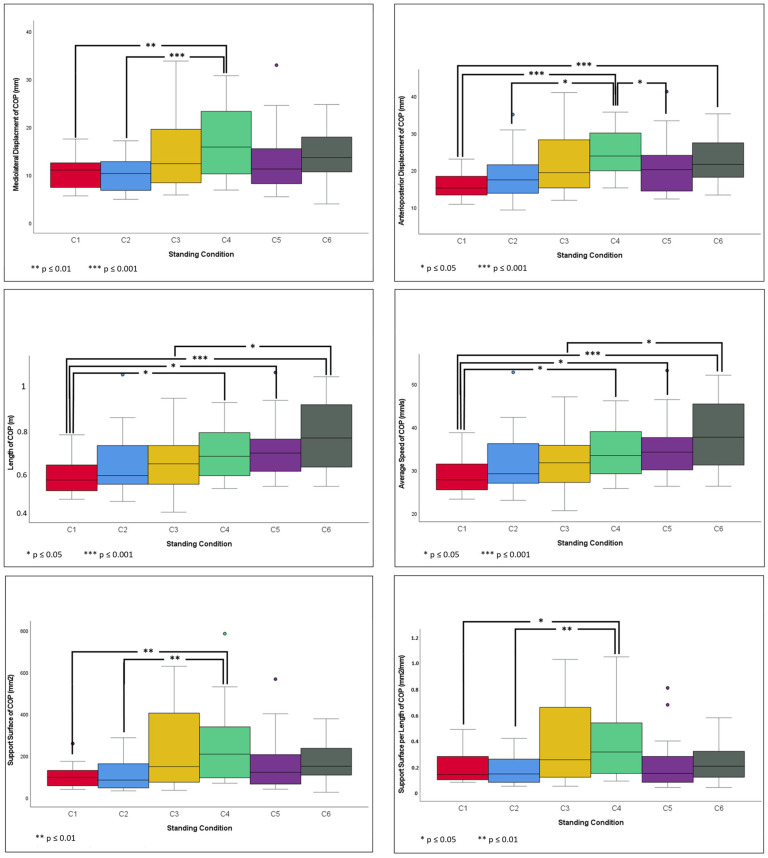
Pairwise comparison of measurement conditions: standing on flat ground with EO (C1), on flat ground with EC (C2), uphill with EO (C3), uphill with EC (C4), downhill with EO (C5), and downhill with EC (C6) for each COP parameter. Connecting lines indicate significant differences among conditions (* *p* ≤ 0.05; ** *p* ≤ 0.01; and *** *p* ≤ 0.001).

Except for the differences between C6 and C1 and between C6 and C3, as previously mentioned, no other differences were observed between C6 and the other remaining conditions. The pairwise comparisons of measurement conditions for each COP parameter are illustrated in Figure 2.

### 3.4. Romberg Index

The mean and standard deviation of the RIs for each COP parameter are summarized in Table 2.

Except for the MLD and LFS, which had values <100, the other COP parameters of RI 1 were higher than 100. However, all COP values of RI 2 and RI 3 exceeded 100. No significant differences were observed among the COP parameters of the RI 1, RI 2, and RI 3 in this study.

## 4. Discussion

This study aimed to evaluate the postural balance of healthy young adults under different aggravating test conditions by challenging visual and somatosensory inputs when standing on steeply inclined and declined surface slopes under both EO and EC conditions. The inclined/declined slope together with the lack of visual input was hypothesized to increase postural instability the most. Increased instability is considered to significantly increase the COP values [9,52] compared with the standard standing condition (C1).

In brief, this study confirms that the most challenging test condition for young adults was standing uphill with EC; all evaluated COP parameters significantly differed from the standard test condition (standing flat with EO). When only the visual input was lost, postural control within the same test condition was not affected.

The study results show that a statistically significant relationship existed between at least two or more COP parameters of C1 and most of the inclined/declined conditions. All the COP parameters between C1 and standing uphill with EC (C4) significantly differed. However, only two COP parameters between C1 and standing downhill with EO (C5) and three COP parameters between C1 and standing downhill with EC (C6) differed significantly in this study. The pairwise comparisons of these significantly different parameters indicated high values for the inclined/declined COP parameters, confirming that the observed values of subjects standing on these sloped surfaces exceeded those of subjects standing on flat ground with EO. Moreover, no significant difference was recorded between C1 and standing uphill with EO (C3). Thus, the hypothesis regarding the relationship between the sloped surface and increase in COP values was partially confirmed.

To assess the vision dependance on postural control, the RI was calculated. No significant difference was recorded between the COP values of EO and EC measurements under each condition (standing on a flat surface, uphill, and downhill). Even though some absolute RI values slightly exceeded the threshold of 100, the absence of vision on its own had no effect on the postural stability when evaluated under the same standing condition. The absence of visual input decreased the postural stability only on the sloped surface in this study. Thus, our second hypothesis regarding the relationship between visual input and postural stability was partially confirmed. These results are in accordance with a recent study among horseback riders, judokas, and young adults where the RI within the static test condition (standing still) remained stable [16]. Even when analyzing RI under distorted visual input when wearing stroboscopic glasses, young adults were able to maintain their balance; however, when an additional sensory input was challenged by standing on a foam pad (somatosensory input), RI indices in all evaluated COP parameters increased [57]. Similar findings were found among young football players; significant increases in conventional COP parameters were observed only when standing on a foam pad in a one-leg stance with EC [54]. These results might indicate that young and healthy adults can compensate for a disturbed/loss of vision when somatosensory and vestibular input remain unaltered by adapting and reweighting the sensory input processing [54,57]. Indeed, it has been shown that the process of reweighting visual and somatosensory inputs reaches optimal performance in adulthood [62].

These findings might be relevant for prophylactic training and rehabilitation purposes in young adults. Optimizing balance skills is an important aspect for many athletes, not only to improve their professional skills but also to prevent injuries. Indeed, it is already known that certain sporting activities influence the mechanisms of balance control to varying degrees [16,54]. Increasing knowledge of sensory control and multisensory reweighting strategies is important to increase our knowledge also in rehabilitation processes and ideally to create (sensory) needs-orientated treatment plans. However, further studies are needed to determine whether balance control mechanisms in young adults are more challenged by somatosensory tasks (e.g., standing on slopes) than by visual tasks (having EC) alone.

The views on the interpretation of COP values reported in the literature vary. The MLD and APD describe the trajectory of the displacement of the COP and subsequently the trajectory of the displacement of the COM in different directions. High MLD and APD values indicate considerable body sway (movement of the COG of the body within the BOS); therefore, postural stability is degraded. The movement of the COP is reported to be slightly greater than the movement of the COG to maintain equilibrium [1]. Note that L is defined as the total length of the displacement of the COP during the observation time. It can be calculated by adding the actual distance of all successive COP locations [1]. In some published articles, the increase in L was reported to be an indicator of decreased postural stability [63,64]; this conclusion was discussed in another report [1]. Palmieri et al. reported that L was the total excursion of the COP path, and a high L value was not necessarily an indicator of decreased postural stability. This was because a high L value could also be observed in a stable stance [1]. A large L value might be an indicator of various small excursions of the COP to achieve stable postural balance [1]. The AS value represents the total distance traveled by the COP during the observation time [1]. An inverse relationship between the AS of the COP and postural stability was reported in the literature [59,65,66]. This conclusion may be disputed because an increase in AS may represent normal active sways of the body to stabilize posture [1]. Therefore, an increase in the AS of the COP may not necessarily indicate a decrease in postural stability.

### 4.1. Flat Surface vs. Sloped Surface

As mentioned, the absence of vision under each standing condition did not affect the COP parameters. In contrast, the comparison between the EC and EO values under various conditions had different results. Despite the increase in the descriptive statistics of C3 (subject standing on a +20° inclined surface with EO) compared with C1, no significant differences were recorded between these two conditions. The moment visual input was missing from the standing uphill condition (C4); all of its COP parameters significantly differed from those of C1. The results of the pairwise comparisons of the COP parameters between C1 and C4 show that C4 had higher COP parameter values. Thus, the COP values under the condition standing uphill with EC exceeded those of a subject standing on flat ground with EO. These findings can be interpreted as follows. Although stability was altered when a subject was standing uphill, it did not significantly affect equilibrium. However, the addition of further disturbances (such as the absence of visual input) significantly increased postural instability. These results are in accordance with previously reported findings in the literature regarding the effect of the absence of visual input on the reduction of balance on sloped surfaces [50,51]. Furthermore, in a study on the electromyogram of leg muscles of subjects standing on different sloped surfaces, the effect of vision on the uphill standing condition was more prominent than on the downhill standing condition [50]. The difference between C1 and C5 (standing on a −20° declined surface condition) was limited to L and AS. The L and AS values indicate that the amplitudes of the MLD and APD did not change in C5; however, L and AS fluctuated at a high frequency. Similar to the standing uphill condition, the absence of vision in the standing downhill condition created another problem for postural stability. Moreover, significantly higher values of APD, L, and AS compared with those in C1 were observed. These results indicate that for young healthy adults, standing uphill with EC is the most difficult condition. This is followed by standing downhill with EC and standing downhill with EO. In terms of MLD, APD, SS, and LFS, C4 was found to be more difficult than C2. The results also show that even in the absence of visual input under both conditions, the body sway of a subject standing uphill increased. Nevertheless, no significant differences were recorded between C2 and C6. Again, this indicates that the absence of visual input when a subject is standing uphill affects the postural stability more significantly than when the subject is standing downhill. In general, in this study, surface slope was observed to have a significant effect on body sway. This observation is the same as those in previous studies. Previous investigations observed higher COP values (L and SS) under inclined/declined standing conditions compared with those when a subject stood on flat ground [51,56,67].

### 4.2. Inclined Slope vs. Declined Slope

Under similar visual conditions (with EC or EO), no significant differences were observed between the uphill and downhill standing conditions in this study. Likewise, no significant difference in terms of L was reported between standing in inclined and declined slopes in the literature [51]. In other words, no significant difference was recorded between the uphill and downhill standing conditions with EO (C3 and C5) and EC (C4 and C6). The differences between standing uphill and downhill conditions with different visual inputs were observed to be limited to L and AS between C3 and C6, and APD between C4 and C5. These findings show that the L and AS under the condition standing downhill with EC were greater than those under the condition standing uphill with EO. However, the APD under the condition standing uphill with EC was higher than that under the condition standing downhill with EO. These results led to the conclusion that the presence or absence of vision is more important than the direction (positive or negative) of the surface slope. This is because between the conditions of standing uphill and downhill, the absence of visual input results in considerable body sways relative to at least one of the COP parameters. These results illustrate the complexity of achieving postural stability and interaction among different systems (such as the visual, somatosensory, vestibular, and central nervous systems) [3]. The active collaboration of these systems ensures that the posture-stabilizing muscles of the legs and trunk respond almost instantaneously to balance disturbances. Therefore, the absence of one of these systems (such as the visual sensory system) or change in one of these systems (such as age-related changes in muscle activity) affects body sway [68,69,70], which may manifest as altered COP values. However, intensifying the balance disturbances by introducing more difficult standing positions (more inclined/declined surfaces) to visual impairment or performing cognitive tasks in altered standing situations might affect the postural balance even more significantly. This is because the attentional demands of concurrent cognitive tasks have a significant impact on postural sway [71].

The effect of surface slope and visual input on postural stability was investigated in previous studies from different points of view [9,50,51,55,56,58,72]. Changes in surface slope angle and visual sense had a significant effect on the length of the APD of COP [51]. The same results were reported for the root mean square of the MLD and APD of COP and sway velocity [58]. Despite similar study aims, the present study differs from the foregoing studies in certain aspects. In terms of sample size, the number of adults in the present study is greater than those in some of the studies [9,50,51]. In one of the studies, only male participants with a wide age range were observed [58]. In terms of the angles of the slopes, the current study was conducted uphill and downhill on +20° and −20° slopes, respectively; these angles were based on the report of Frames et al. in 2013 [9]. In contrast, the other studies used different slopes [50,51,56,58,67]. Furthermore, the number of COP parameters in the present study (six parameters) exceeds those in other studies.

### 4.3. Effect of BMI and Gender

As reported in [34,36,37,39,73,74], anthropometric parameters affect postural stability. Height [37,39,73], body mass [34,37,73], body fat [39], BMI [74], and obesity [35,38,40,75] are the important anthropometric parameters discussed in the literature; they are reported to be correlated with postural balance. To evaluate the effect of anthropometric parameters on COP values, BMI was calculated in this study. No significant relationship was recorded between the MLD and SS of the COP and BMI; however, the APD of the COP had a significant relationship with the BMI. These findings confirm the effect of anthropometric values on postural sway. Due to the nature of the upright bipedal standing position and BOS, the posture of participants was more unstable in the anterior–posterior direction than in the mediolateral [17,76]. Note that these results were those of healthy adults; increased age or pathologic conditions could change these results or render the MLD and SS significant. Parameters L, AS, and LFS were also found to be related to the BMI. Of note, the potential bias introduced by variations in height (SD = 11 cm) and weight (SD = 17.7 kg) among the study participants, both affecting BMI, must be acknowledged. Interestingly, weight is reported to be a more important anthropometric factor than height [34,37]. Nevertheless, the effect of height on equilibrium cannot be ignored. Increased body height was reported to worsen the postural balance [36,37,39]. Most recently, a positive effect on balance control was demonstrated when participants bent their knees [77]. It is thought that the suspensory strategy, which involves lowering the COM in a vertical direction towards the BOS, changes the sensory integration of the systems involved in maintaining balance and thus leads to improved postural stability [77]. We suggest that future studies should include a study population with more coherent anthropometric parameters to better disentangle the effects of weight, height, and BMI on balance.

The relationship between the gender of participants and COP values was also investigated. Similar to the previously reported results for older adults at the time of retirement [78], no significant association was found between the gender and COP values in the current study. These findings differed from those reported by Kim et al. (2012) for young adults [37]. The number of participants may influence the results because the two foregoing studies had a larger sample size than the present study. Hence, a small sample size can be considered as a limitation of this study. In interpreting the results, consider that a relatively small number of subjects (11 females and 11 males) with an age range of 24–34 years old was used in this study. Increasing the sample size by including other age groups (young and old adults) and people with different health conditions may affect the results and lead to a more definitive conclusion in future research.

In our study, we focused on examining linear COP parameters. To enhance future investigations, we suggest employing non-linear parameters like sample entropy. This method enables the assessment of the non-linearity within postural sway dynamics by evaluating the irregularities present in the COP’s time series data [16]. Integrating such non-linear measures could provide a more comprehensive understanding of the complexity of postural control.

## 5. Conclusions

In conclusion, significant differences were observed between the standing conditions and COP parameters in this study. Most of the differences were observed between standing on a flat surface with EO and standing uphill with EC (C4). Therefore, the most difficult standing condition for the participants in this study was C4. No differences were recorded under the same standing conditions with EO and EC. When a subject stood on an inclined surface, the loss of vision significantly increased the postural instability. However, the posture of subject standing uphill with EC was more unstable than that of a subject standing downhill. Furthermore, the presence or absence of visual sense while standing on sloped surfaces was more important than the direction of the slope (uphill or downhill). Postural stability was demonstrated to be affected by BMI in this study. In contrast, no relationship was found between the COP parameters and gender of participants. Therefore, any alteration in visual or somatosensory inputs can affect postural stability; however, a combination of these restrictions can affect equilibrium more efficiently.

## Figures and Tables

**Table 1:** Mean and standard deviation of COP parameters under different standing conditions.

Standing Condition	COP Parameter (Mean ± SD)					
	MLD (mm)	APD (mm)	L (m)	AS (mm/s)	SS (mm^2^)	LFS (mm^2^/mm)
C1	10.57 ± 3.64 #	16.32 ± 3.64 #§	0.58 ± 0.08 #†§	28.84 ± 4.25 #†§	111.07 ± 61.97 #	0.20 ± 0.12 #
C2	9.93 ± 3.65 †	18.70 ± 6.78 †	0.64 ± 0.15	32.12 ± 7.33	109.88 ± 72.13 †	0.18 ± 0.12 †
C3	15.26 ± 8.05	21.98 ± 8.52	0.64 ± 0.12 ¥	31.94 ± 5.95 ¥	230.61 ± 193.20	0.37 ± 0.31
C4	16.55 ± 7.08 #†	24.93 ± 6.62 #†¥	0.68 ± 0.12 #	34.11 ± 6.07 #	240.56 ± 173.88 #†	0.35 ± 0.24 #†
C5	12.68 ± 6.52	20.33 ± 7.24 ¥	0.80 ± 0.36 †	39.79 ± 18.12 †	160.03 ± 131.60	0.23 ± 0.20
C6	14.19 ± 5.44	22.38 ± 5.98 §	0.80 ± 0.25 §¥	39.94 ± 12.31 §¥	179.97 ± 102.46	0.25 ± 0.17

#, †, §, ¥: Mean values in each column (e.g., MLD, APD) with identical symbols had significant differences in terms of standing conditions (*p* < 0.05). For instance, in the MLD column, there is a significant difference between C1 and C4, as indicated by #. Additionally, there is a significant difference between C2 and C4, as indicated by †. MLD: mediolateral displacement; APD: anterior–posterior displacement; L: length of COP; AS: average speed of COP; SS: support surface of COP; LFS: support surface per length of COP; C1: standing on flat ground with EO; C2: standing on flat ground with EC; C3: standing uphill with EO; C4; standing uphill with EC; C5: standing downhill with EO; and C6: standing downhill with EC.

**Table 2:** Mean and standard deviation of RIs for each COP parameter.

RI	COP Parameter (Mean ± SD)					
	MLD (mm)	APD (mm)	L (m)	AS (mm/s)	SS (mm^2^)	LFS (mm^2^/mm)
RI 1	95.52 ± 22.12	114.0 ± 27.91	110.58 ± 14.80	110.87 ± 14.79	100.87 ± 48.0	94.13 ± 49.79
RI 2	121.75 ± 59.81	122.04 ± 36.53	107.60 ± 10.39	107.42 ± 10.24	147.25 ± 114.47	135.33 ± 101.31
RI 3	122.65 ± 47.23	116.03 ± 31.31	106.24 ± 26.02	106.35 ± 26.01	139.78 ± 76.55	130.84 ± 56.77

RI: Romberg index; MLD: mediolateral displacement; APD: anterior–posterior displacement; L: length of COP; AS: average speed of COP; SS: support surface of COP; and LFS: support surface per length of COP.

## Data Availability

The raw data supporting the findings of this article will be made available by the authors, on reasonable request.

## Supplementary Materials

The following supporting information can be downloaded at https://www.mdpi.com/article/10.3390/life14020227/s1, Table S1: Detailed results of the pairwise comparisons between the standing condition and COP parameters with Bonferroni’s alpha corrections.

## Abbreviations

The article contains the following abbreviations.

EO	eyes open
EC	eyes closed
COP	center of pressure
COM	center of mass
BOS	base of support
COG	center of gravity
BMI	Body Mass Index
MLD	mediolateral displacement
APD	anterior–posterior displacement
L	total length
AS	average speed
SS	support surface
LFS	quotient of support surface in square millimeters per unit length
C1	standing on flat ground with EO (standard condition)
C2	standing on flat ground with EC
C3	standing uphill with EO
C4	standing uphill with EC
C5	standing downhill with EO
C6	standing downhill with EC
RI	Romberg Index
