# Supplementary material for: Effect of Vision and Surface Slope on Postural Sway in Healthy Adults: A Prospective Cohort Study
Source: Life (Basel). 2024 Feb 5;14(2):227. doi: 10.3390/life14020227 (PMC10890291; doi:10.3390/life14020227)
Supplement: Supplementary file 1 [file life-14-00227-s001.zip › life-2840593-supplementary.docx]

Supplementary material

**Table S1.** Detailed results of the pairwise comparisons between the standing condition and COP parameters with Bonferroni’s alpha corrections.

| **Standing**  **Condition** | | **MLD** | | | **APD** | | | **L** | | | **AS** | | | **SS** | | | **LFS** | | |
| --- | --- | --- | --- | --- | --- | --- | --- | --- | --- | --- | --- | --- | --- | --- | --- | --- | --- | --- | --- |
| I | J | Mean  Difference (I−J) | Std. Error | p−value | Mean  Difference (I−J) | Std. Error | p−value | Mean  Difference (I−J) | Std. Error | p−value | Mean  Difference (I−J) | Std. Error | p−value | Mean  Difference (I−J) | Std. Error | p−value | Mean  Difference (I−J) | Std. Error | p−value |
| C1 | C2 | 0.03 | 0.04 | 1.00 | −0.04 | 0.04 | 1.00 | −0.04 | 0.02 | 1.00 | −0.04 | 0.02 | 1.00 | 0.04 | 0.08 | 1.00 | 0.04 | 0.08 | 1.00 |
|  | C3 | −0.13 | 0.06 | 0.48 | −0.11 | 0.04 | 0.11 | −0.04 | 0.02 | 0.65 | −0.04 | 0.02 | 0.58 | −0.21 | 0.09 | 0.44 | −0.21 | 0.09 | 1.00 |
|  | C4 | −0.181* | 0.05 | 0.01 | −0.179* | 0.03 | 0.00 | −0.070* | 0.02 | 0.02 | −0.071* | 0.02 | 0.02 | −0.296* | 0.07 | 0.00 | −0.296* | 0.07 | 0.05 |
|  | C5 | −0.06 | 0.05 | 1.00 | −0.08 | 0.03 | 0.11 | −0.115* | 0.03 | 0.02 | −0.116* | 0.03 | 0.02 | −0.10 | 0.07 | 1.00 | −0.10 | 0.07 | 1.00 |
|  | C6 | −0.12 | 0.05 | 0.26 | −0.132* | 0.03 | 0.00 | −0.129* | 0.03 | 0.00 | −0.130* | 0.03 | 0.00 | −0.18 | 0.07 | 0.24 | −0.18 | 0.07 | 1.00 |
| C2 | C3 | −0.16 | 0.06 | 0.15 | −0.07 | 0.05 | 1.00 | 0.00 | 0.02 | 1.00 | 0.00 | 0.02 | 1.00 | −0.25 | 0.10 | 0.25 | −0.25 | 0.10 | 0.25 |
|  | C4 | −0.212* | 0.05 | 0.00 | −0.135* | 0.04 | 0.01 | −0.03 | 0.02 | 1.00 | −0.03 | 0.02 | 1.00 | −0.335* | 0.08 | 0.00 | −0.335* | 0.08 | 0.01 |
|  | C5 | −0.09 | 0.05 | 1.00 | −0.04 | 0.04 | 1.00 | −0.08 | 0.04 | 0.58 | −0.07 | 0.04 | 0.61 | −0.14 | 0.08 | 1.00 | −0.14 | 0.08 | 1.00 |
|  | C6 | −0.15 | 0.05 | 0.06 | −0.09 | 0.04 | 0.37 | −0.09 | 0.03 | 0.08 | −0.09 | 0.03 | 0.08 | −0.22 | 0.08 | 0.14 | −0.22 | 0.08 | 1.00 |
| C3 | C4 | −0.05 | 0.06 | 1.00 | −0.07 | 0.04 | 1.00 | −0.03 | 0.02 | 1.00 | −0.03 | 0.02 | 1.00 | −0.08 | 0.10 | 1.00 | −0.08 | 0.10 | 1.00 |
|  | C5 | 0.07 | 0.06 | 1.00 | 0.03 | 0.04 | 1.00 | −0.08 | 0.03 | 0.40 | −0.07 | 0.03 | 0.42 | 0.12 | 0.10 | 1.00 | 0.12 | 0.10 | 0.94 |
|  | C6 | 0.01 | 0.06 | 1.00 | −0.02 | 0.04 | 1.00 | −0.089* | 0.03 | 0.03 | −0.088* | 0.03 | 0.03 | 0.03 | 0.10 | 1.00 | 0.03 | 0.10 | 1.00 |
| C4 | C5 | 0.12 | 0.05 | 0.36 | 0.097* | 0.03 | 0.05 | −0.05 | 0.03 | 1.00 | −0.05 | 0.03 | 1.00 | 0.20 | 0.08 | 0.23 | 0.20 | 0.08 | 0.06 |
|  | C6 | 0.06 | 0.05 | 1.00 | 0.05 | 0.03 | 1.00 | −0.06 | 0.03 | 0.54 | −0.06 | 0.03 | 0.53 | 0.12 | 0.08 | 1.00 | 0.12 | 0.08 | 0.71 |
| C5 | C6 | −0.06 | 0.05 | 1.00 | −0.05 | 0.03 | 1.00 | −0.01 | 0.04 | 1.00 | −0.01 | 0.04 | 1.00 | −0.08 | 0.08 | 1.00 | −0.08 | 0.08 | 1.00 |

MLD: mediolateral displacement; APD: anterior–posterior displacement; L: length of COP; AS: average speed of COP; SS: support surface of COP; LFS: support surface per length of COP; C1: standing on flat ground with EO; C2: standing on flat ground with EC; C3: standing uphill with EO; C4; standing uphill with EC; C5: standing downhill with EO; and C6: standing downhill with EC. * The mean difference is significant at the 0.05 level.
